# High Serum Levels of HDV RNA Are Predictors of Cirrhosis and Liver Cancer in Patients with Chronic Hepatitis Delta

**DOI:** 10.1371/journal.pone.0092062

**Published:** 2014-03-21

**Authors:** Raffaella Romeo, Barbara Foglieni, Giovanni Casazza, Marta Spreafico, Massimo Colombo, Daniele Prati

**Affiliations:** 1 AM & A Migliavacca Center for Liver Disease, 1st Division of Gastroenterology, IRCCS Fondazione Cà Granda Ospedale Maggiore Policlinico, University of Milan, Milan, Italy; 2 Department of Transfusion Medicine and Hematology, Ospedale A. Manzoni, Lecco, Italy; 3 Dipartimento di Scienze Biomediche e Cliniche “L. Sacco”, University of Milan, Milan, Italy; Hannover Medical School, Germany

## Abstract

Chronic infection with the hepatitis delta virus (HDV) is a risk factor for cirrhosis and hepatocellular carcinoma (HCC), but little is known whether the outcome of hepatitis is predicted by serum markers of HDV and hepatitis B virus (HBV) infection. The aim of the study was to investigate these correlations in 193 patients with chronic HDV infection who had been followed up for a median of 9.5 years (4.8–19.3). HDV-RNA was first measured by qualitative in-house nested RT-PCR and quantified by in-house real-time PCR. HDV RNA levels only appeared significantly associated to HCC (univariate analysis: OR 1.32, 95% CI 1.02–1.71; p = 0.037; multivariate analysis: OR 1.42, 95% CI 1.04–1.95; p = 0.03). In non-cirrhotics at first presentation (n = 105), HDV RNA levels were associated with progression to cirrhosis (univariate analysis: OR = 1.57, 95% CI 1.20–2.05, p<0.001; multivariate analysis: OR = 1.60, 95% CI 1.20–2.12, p = 0.007) and development of HCC (univariate analysis: OR = 1.66, 95% CI 1.04–2.65, p = 0.033; multivariate analysis: OR = 1.88, 95% CI 1.11–3.19, p = 0.019). ROC analysis showed that approximately 600,000 HDV RNA copies/mL was the optimal cut-off value in our cohort of patients for discriminating the development of cirrhosis. High levels of HDV viremia in non-cirrhotic patients are associated with a considerable likelihood of progression to cirrhosis and the development of HCC. Once cirrhosis has developed, the role of HDV replication as a predictor of a negative outcome lessens.

## Introduction

Hepatitis delta virus (HDV) is a unique agent characterised by a single-stranded RNA genome encapsidated by the hepatitis B surface antigen (HBsAg) and a peculiar strategy of infection of the target organ [1]. In fact, HDV requires the helper functions provided by hepatitis B virus (HBV) in order to propagate to hepatocytes, it can only infect subjects with co-existing HBV infection due either to the simultaneous transmission of the two viruses or superinfection in an established HBV carrier [2], [3]. Not surprisingly therefore, the incidence of HDV infection declined rapidly in Southern Europe and the Mediterranean basin after the early 1990s following the introduction of HBV vaccinations [4], but the many immigrants from highly endemic areas carrying chronic HDV infection has led to the emergence of a new scenario in Western Europe as a whole [5]–[7].

Chronic HDV infection has always been considered severe and rapidly progressive, leading to end-stage liver disease in just a few years, whereas a more slowly progressive form has been identified which evolved into cirrhosis in 30% of the patients followed up for periods of up to 28 years, with liver decompensation rather than liver cancer (HCC) being the first cirrhosis-related complication to appear [8]. These observations suggest the existence of patient populations with heterogeneously evolving forms of chronic HDV infection, but the pathogenesis of HDV-related liver damage is still controversial. It seems unlikely that the virus has a direct cytopathic effect because studies have found liver grafts expressing HDAg alone without any tissue injury [9]. On the other hand, the so-called replication-associated cytopathogenicity of the virus might explain the tissue damage observed in patients with acute HDV infection, which is characterised by high levels of viral replication, whereas chronic infection is characterised by low levels of viral production and pathogenicity [2], [10]. Furthermore, recent findings indicating the existence of a direct correlation between serum levels of HBsAg and HDV RNA in the absence of an inverse correlation with HBV DNA levels suggests an alternative interpretation of HDV pathogenicity that is more closely related to HBV biology [11].

The aim of this study was to investigate the correlations between viremia (serum HBsAg, HBV DNA and HDV RNA levels) and the clinical outcomes of chronic HDV infection, including the progression to cirrhosis, the development of HCC, decompensation, and liver-related death.

## Patients and Methods

### Patients

Patients were selected from the previously published cohort attending the Liver Centre of Ospedale Maggiore in Milan (Italy) [8]. The only selection criteria was the availability of multiple frozen serum samples (−70°C) collected at first visit at our division (baseline sample), as well as at least one frozen sample collected over follow-up. The study was approved by the Institutional Review Board of the Department of Internal Medicine at Maggiore Hospital. Patients gave their written informed consent to take blood for experiments and gave permission for use of their medical records.

### Diagnosis of HDV Infection

HDV infection was defined as the presence of HDV antigen (HDAg) in liver tissue, or the detection of serum HDV RNA in anti-HDV/HBsAg seropositive patients as detailed elsewhere [12].

### HDV RNA Extraction

HDV RNA was extracted from 200 μL of plasma using the High Pure Viral Nucleic Acid kit (Roche Applied Science, Basel, Switzerland). As many of the samples had very low concentrations of HDV RNA, it was essential to ensure efficient viral nucleic acid extraction. We therefore modified the extraction protocol including a preliminary step of one hour’s incubation with the lysis/binding buffer supplied with the kit (chaotropic salt, poly(A), and proteinase K) at room temperature.

### Quantitative HDV RNA Real-time PCR

An HDV RNA reference standard for the quantification of HDV RNA by Real Time PCR is not currently available. A 569 bp fragment of the HDV genome including the 5′ UTR HDV region has been cloned into a plasmid, which was used as positive control transcript for HDV Real Time PCR assay. Alignment of full-length HDV GenBank reference genomes representative of all HDV genotypes was used to locate conserved regions suitable for HDV RNA amplification, and the 5′ untranslated (UTR) region was identified as the most appropriate, as defined by others [13]. Thus, primers and probes were designed to amplify a 110 bp fragment of the 5′ UTR, taking into account the genetic variability of HDV genotypes. The assay was based on the use of fluorescence resonance energy transfer (FRET) probes, and included the forward primer HDV-s AGTGGCTCTCCCTTAGCCAT, the reverse primer HDV-as CGTCCTTCTTTCCTCTTCGGGT, the 3′-fluorescein-labelled probe HDV-FL TCCTCCTTCGGATGCCCAGGTCG, and the 5′-LC640-labelled probe HDV-LC CCGCGAGGAGGTGGAGATGCCAT. The reaction was performed using a LightCycler 2.0 instrument (Roche Diagnostics) and a 20 μL total volume containing 1× LightCycler RNA Master Hybridization Probe, 55 nM Mn(OAC)_2_, 10 μM of each primer and probe, and 5 μL of RNA, and consisted of initial RNA retrotranscription (and Taq activation) for 25′ at 61°C followed by 30″ at 95°C, and then 45 amplification cycles (1″ at 95°C, 5″ at 62°C, and 4″ at 72°C) and a final melting curve analysis (30″ at 72°C, 20″ at 95°C, 30″ at 40°C, 1″ at 85°C, and 30″ at 40°C). All quantification were performed relatively to the plasmid standard described, which was used to define the 4-point quantification curve in each assay. Moreover, a real sample from the same patient was introduced in each Reverse Transcription-Real Time PCR analysis as positive internal control. The linearity of the PCR ranged from 10^3^ to 10^8^ copies/mL, and the sensitivity was 500 copies/mL. All samples were tested in duplicate and the mean of the two analysis was considered for statistical analysis. For the validation of the assay, a subset of 50 samples were also tested with a commercial CE IVD HVD Real Time PCR assay (LifeRiver Diagnostics, Shanghai, China), with concordant results (data not shown).

### HBsAg Quantification

HBsAg was quantified by means of automated chemiluminescent microparticle immunoassays (Architect HBsAg, Abbott, Chicago, IL).

### HBV DNA Quantification

For the purposes of this study, all of the patients were re-tested for HBV DNA by means of quantitative real-time PCR using a COBAS AmpliPrep/COBAS TaqMan automated system (HBV Test, Roche Diagnostics).

### Liver Biopsy

Liver biopsies were obtained using a Tru-cut needle (16 gauge, Travenol, Hyland, CA; Uro-Cut 16G, TSK, Tokyo, Japan), and the sections were stained by means of conventional procedures. Liver cell necrosis, inflammation and fibrosis were graded on the basis of the METAVIR scoring system [14].

### Disease Progression

Progression to cirrhosis in patients presenting with chronic hepatitis at entry was defined on the basis of fibrosis at liver histology (F 4) [14], a platelet count of <100,000 mm^3^, the ultrasound (US) features of surface nodularity, splenomegaly, a portal vein diameter of >15 mm, endoscopically revealed oesophageal varices.

In the patients presenting with cirrhosis at entry, disease progression was defined as entry into a higher Child-Pugh class, and the development of clinical decompensation or HCC [15].

All of the patients with a clinical or histological diagnosis of cirrhosis underwent US surveillance and serum AFP determinations every 6 months as previously described [16]. HCC was diagnosed on the basis of the criteria used at the time of patient evaluation; since 2001, we used the guidelines published by the European Association for the Study of the Liver (EASL) [17]. In patients undergoing echo-guided liver biopsy, HCC was classified using internationally accepted criteria based on the pattern of invasive or replacing growth [18]. Complications of cirrhosis other than HCC, such as ascites, jaundice, hepatic encephalopathy and GI bleeding, were diagnosed and treated in accordance with established criteria [19], .

### Statistical Analysis

The categorical variables are given as numbers and percentages, and the continuous variables as mean values±standard deviation or median values and range, as appropriate. The serological variables were log-transformed (base 10 logarithm) before analysis. The chi-squared test and t test for independent samples were respectively used to compare proportions and mean values. The non parametric Mann–Whitney test was used in case of variables with non normal distributions. Univariate logistic regression models were used to assess the effect of the serological variables at the time of presentation and the risk of developing an event of interest (cirrhosis, HCC, liver decompensation, liver-related death, and “at least one of these”) during the follow-up. In addition, multivariate logistic regression analyses were performed including in the above described univariate models some potential relevant confounders (age, sex, alcohol consumption, HBeAg and IFN). Finally, a subgroup analysis was performed to assess the effect of the variables of interest separately on the two subgroups of patients defined according to the clinical diagnosis at baseline (chronic hepatitis or cirrhosis). Odds ratios (ORs) with their 95% confidence intervals were calculated for the variables included in the models. In the logistic models, only patients with an available serological determination before the development of the event were included. A receiver operating characteristic (ROC) analysis was used to asses the ability of log-transformed HDV RNA to predict the development of cirrhosis in patients with chronic hepatitis. The area under the curve (AUC) was calculated as an overall assessment of the predictive ability of HDV RNA.

The sensitivity, specificity, positive predictive value (PPV) and negative predictive value (NPV) of the measurements were calculated, and the optimal HDV RNA cut-off value for predicting the development of cirrhosis was chosen on the basis of the maximum Youden index (the sum of sensitivity and specificity minus one). P values of <0.05 (two-tailed) were considered statistically significant. All of the statistical analyses were made using SAS statistical software (release 9.2) (SAS Institute Inc., Cary, NC, USA).

## Results

We selected 193 patients for whom a baseline serum sample was available, as well as at least one sample collected over follow-up, with a median of three (2–6) samples/patient. We were able to select an homogeneous group of patients that reflected the original population of 299 patients. Indeed, among the 193 cases selected for the present study (65% of the original population), there were 35 patients who developed HCC (46 in the original cohort) and 35 who experienced liver decompensation (54 in the original cohort).

Table 1 shows the main epidemiological and virological characteristics of the 193 patients at the time of study entry, i.e. at the diagnosis of HDV infection, as compared to the general cohort of 299 patients [8], that also included patients who were diagnosed to have HDV infection in other institutions.,. The patients were prevalently Italians (94%), males (76%) with a mean age of 41 years. The modality of infection was sporadic in the vast majority of cases (146/193, 76%), and 15 patients (8%) were HBeAg positive. Thirty-eight cases (20%) had a previous history of an alcohol intake of >40 g/day. HBsAg could be quantified in 187 cases (97%), and HBV DNA was detectable in 169 cases (87%). All of the samples were HDV RNA positive upon qualitative testing, and viremia could be quantified in 157 samples (81%); the remaining 36 samples (19%) had concentrations that were below the detection limit of the quantitative assay. Liver cirrhosis was diagnosed at baseline in 88 cases (45%) by histological criteria in 75 (85%) and clinical criteria in the remaining 13 (15%). The proportion of cirrhosis was slightly higher in the current cohort because we are a referral centre for both HBV infection and advanced liver diseases. Therefore, we often received from other Italian institutions patients with HBV related cirrhosis before the diagnosis of HDV coinfection was made. The clinical criteria for diagnosing cirrhosis were platelets below 100.000 mm^3^ in all the 13 patients, the ultrasound (US) features of surface nodularity in all patients, splenomegaly in eight, a portal vein diameter of >15 mm in five, presence of oesophageal varices in three. 82 (93%) patients were Child-Pugh class A, six (7%) were Child-Pugh class B.

**Table 1:** Comparison of the main epidemiological and virological characteristics of the 193 patients (current cohort) and 299 patients (general cohort [8]) at study entry.

	Current Cohort	General Cohort	
	(n° = 193)	(n° = 299)	p
Italian origin, n° (%)	181 (94)	290 (97)	ns
Males, n° (%)	147 (76)	230 (77)	ns
Mean age, years	40.8	30.5	na
Modality of infection, n° (%)			
Unknown	146 (76)	222 (74)	ns
Post-transfusion	14 (7)	27 (9)	ns
Intra venous drug use	24 (12)	38 (13)	ns
Sexual activity	9 (5)	12 (4)	ns
Alcohol >40 g/day, n° (%)	38 (20)	69 (23)	ns
HBsAg quantified, n° (%)	187 (97)	nd	
HBeAg +, n° (%)	15 (8)	27 (9)	ns
HDV-RNA positive, n° (%)	193 (100)	299 (100)	ns
HDV-RNA >500 cp/mL, n° (%)	157 (81)	nd	
HBV DNA >50 IU/mL, n° (%)	169 (87)	nd	
Liver cirrhosis, n° (%)	88 (45)	104 (35)	= 0.03*
Median follow-up, years	9.5 (4.8–19.3)	21 (1.2–43)	na

nd = not done. In the general cohort [8] HBsAg, HDV RNA and HBV DNA were not quantified.

na = not applicable. In the original cohort [8], mean age and median follow-up were calculated from the first evidence of chronic liver disease. In the current cohort, mean age and follow-up were calculated at entry into the current study, corresponding to first access to our unit as well as time of collection of the first serum sample available for testing.

*The proportion of cirrhosis was slightly higher in the current cohort because we are a referral centre for both HBV infection and advanced liver diseases. Therefore, we often received from other Italian institutions patients with HBV related cirrhosis before the diagnosis of HDV coinfection was made.

We were not able to evaluate differences between the two cohorts in terms viral load, since baseline levels of HBsAg, HDV RNA and HBV DNA could not be quantified in the general cohort. Finally, in the original cohort, mean age and median follow-up were calculated from the first evidence of chronic liver disease, while in the current cohort, mean age and follow-up were calculated at entry into the current study, corresponding to first access to our unit, when the first serum sample was stored.

Table 2 shows the main characteristics of the patients with and without liver cirrhosis. The patients with chronic hepatitis were significantly younger than those with cirrhosis (37.3±11.0 *vs* 44.9±10.3, p<0.001), had different modalities of infection (p = 0.024) and longer follow-up (p = 0.0003). There were no differences in terms of gender, alcohol intake, or baseline levels of HBsAg, HBV DNA or HDV RNA.

**Table 2:** Main baseline epidemiological and virological characteristics of the patients with and without cirrhosis.

	Chronic hepatitis	Cirrhosis	
	(n = 105)	(n = 88)	p
Males, No.	83 (79%)	64 (73%)	ns
Mean age ± SD, years	37.3±11.0	44.9±10.3	<0.001
Modality of infection, No.			p = 0.0236
Unknown	71 (68%)	75 (85%)	
Post-transfusion	9 (8%)	5 (6%)	
Intra venous drug use	17 (16%)	7 (8%)	
Sexual activity	8 (8%)	1 (1%)	
Median follow-up, years	12.5 (6.6–19.9)	6.8 (3.8–16.5)	p = 0.0003
Alcohol >40 g/day, No.	23 (22%)	15 (17%)	ns
Mean log HBsAg ± SD	3.4±0.8	3.6±0.6	ns
Mean log HBV-DNA ± SD	3.0±1.6	3.0±1.4	ns
Mean log HDV-RNA ± SD	5.0±1.9	5.2±1.6	ns
IFN treatment, No	51 (48%)	37 (42%)	ns
Virological Response, No	18 (17%)	15 (17%)	ns

88 patients (45%) were previously treated with standard Interferon at doses between 6 and 9 MU, for a median of 18 months (6–94). 51/88 had chronic hepatitis at baseline but 20 of them progressed to cirrhosis over follow-up (histological diagnosis in all cases), after a median treatment of 24 months (6–56). 33/88 had a virological response (i.e. persistently negative serum HDV RNA or liver HDAg in at least 2-point analysis during 3 years of follow-up,18 with chronic hepatitis). However, in spite of the virological response, 5 chronic hepatitis progressed to cirrhosis documented at liver histology.

Considering the occurrence of HCC, liver decompensation and liver related death as clinical endpoints of the study, we performed the univariate and multivariate analysis of the principal epidemiological, clinical and virological variables. Among the 193 patients at baseline, 28 (14.5%) developed HCC, 26 (13.5%) developed liver decompensation and 25 (13%) died of liver related events. By univariate analysis, the virological variables only emerged as associated to the clinical outcome (Table 3). In particular, HDV RNA levels appeared significantly associated to HCC development (OR 1.32, 95% CI 1.02–1.71; p = 0.037) and occurrence of at least one unfavourable event (i.e. HCC, liver decompensation, liver related death) (OR 1.36, 95% CI 1.10–1.68; p = 0.004). Levels of HBsAg were significantly associated to the occurrence of liver decompensation (OR 2.31, 95% CI 1.09–4.91; p = 0.03). However, by multivariate analysis, serum HDV RNA levels only were confirmed as associated to HCC development (OR 1.42, 95% CI 1.04–1.95; p = 0.03) and occurrence of at least one unfavourable outcome (OR 1.41, 95% CI 0.99–3.21; p = 0.007).

**Table 3:** Results of univariate and multivariate analyses according to clinical outcomes in the 193 patients.

	HCC				Liver decompensation				Liver-related death		At least 1 event			
	(28 patients)				(26 patients)				(25 patients)		(48 patients)			
	Univariate		Multivariate		Univariate		Multivariate		Univariate		Univariate		Multivariate	
	OR	p	OR	p	OR	p	OR	p	OR	p	OR	p	OR	p
**HDV-RNA**	1.32	0.037	1.42	0.026	1.26	0.076	1.12	0.140	1.16	0.251	1.36	0.004	1.41	0.007
	(1.02–1.71)		(1.04–1.95)		(0.98–1.63)		(1.11–3.19)		(0.95–2.86)		(1.10–1.68)		(0.99–3.21)	
**HBV-DNA**	1.0	0.961	0.98	0.903	1.23	0.081	1.16	0.233	1.21	0. 131	1.14	0.199	1.14	0.293
	(0.78–1.30)		(0.71–1.35)		(0.97–1.56)		(0.64–1.65)		(0.58–1.56)		(0.93–1.39)		(0.72–2.09)	
**HBsAg** *	1.29	0.440	0.90	0.811	2.31	0.029	1.38	0.233	1.83	0.099	1.74	0.052	1.73	0. 678
	(0.68–2.46)		(0.38–2.13)		(1.09–4.91)		(0.19–1.38)		(0.36–1.73)		(0.99–3.06)		(0.13–1.05)	

Multivariate models included HDV-RNA, HBV-DNA, HBsAg, age, sex, alcohol consumption, HBeAg and IFN.

*Seven patients with missing values.

**Figure 1:**
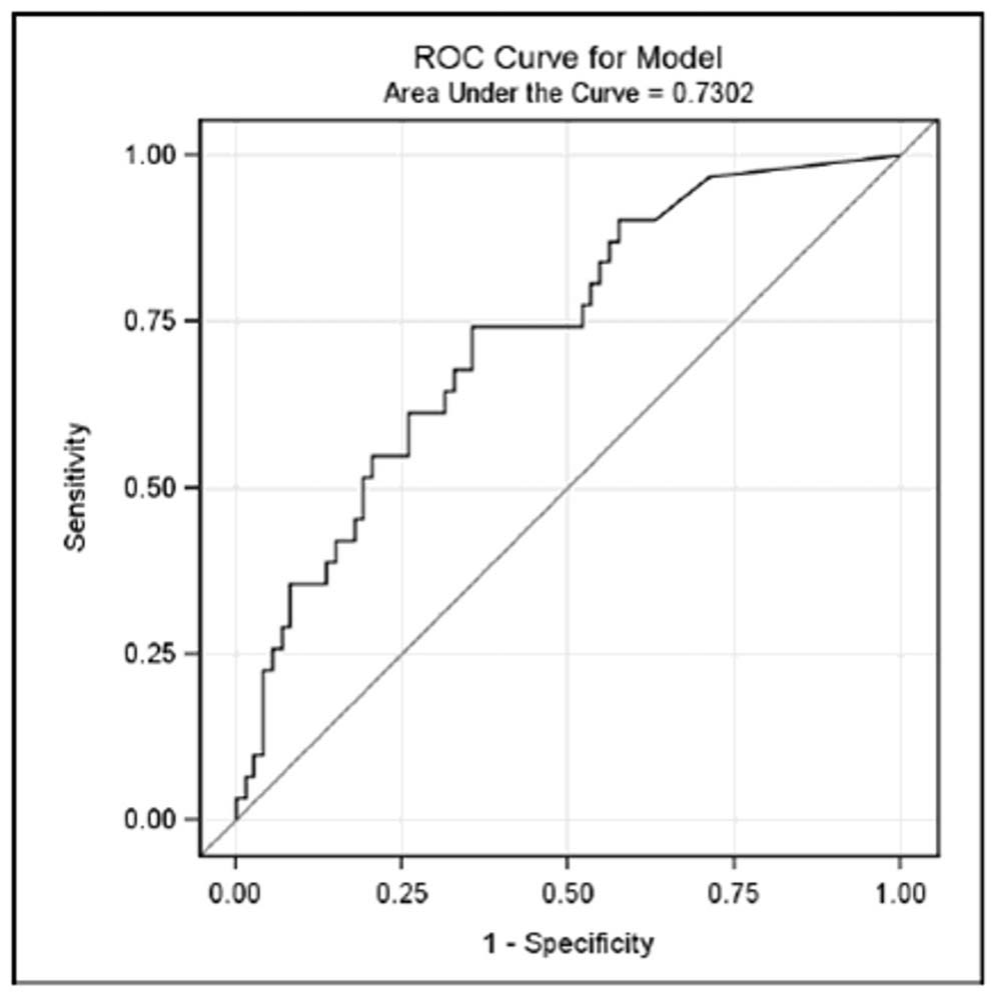
ROC analysis of HDV RNA levels. With a view to identifying levels of HDV viremia correlated to a higher propensity of disease progression, the ROC analysis identified 5.78 logHDV RNA (i.e. approximately 600,000 copies/mL) as the best cut-off value for predicting the development of cirrhosis (AUC = 0.73) in patients with chronic hepatitis.

We also performed univariate and multivariate analysis separately on patients with chronic hepatitis as well as in cirrhotic patients. Among the 105 patients with chronic hepatitis at baseline, 31 (29%) developed cirrhosis, 10 (9%) developed HCC, 7 (6%) developed liver decompensation and 7 (6%) died of liver related events. The results of the univariate analyses of patients with chronic hepatitis at baseline are summarised in Table 4. Univariate analysis showed that the baseline HDV RNA levels of the patients with chronic hepatitis were associated with progression to cirrhosis (OR = 1.57, 95% CI 1.20–2.05, p<0.001) and the development of HCC (OR = 1.66, 95% CI 1.04–2.65, p = 0.033). These findings were confirmed by the multivariate analysis (cirrhosis: OR = 1.56, 95% CI 1.14–2.12; p = 0.005; HCC: OR = 2.64, 95% CI 1.13–6.16; p = 0.025). Among the 88 patients with cirrhosis at baseline, 25 (28%) developed HCC, 28 (32%) developed liver decompensation and 28 (32%) died of liver related death. Univariate analysis showed no association between the serological parameters and the risk of any of the unfavourable events. Finally, with a view to identifying levels of HDV viremia correlated to a higher propensity of disease progression, we performed the ROC analysis in patients with chronic hepatitis. The ROC analysis identified 5.78 logHDV RNA (i.e. approximately 600,000 copies/mL) as the best cut-off value for predicting the development of cirrhosis (AUC = 0.73) in patients with chronic hepatitis (Fig. 1). This value was calculated in our patient cohort and with our HDV RNA quantification methods. This threshold had a sensitivity of 74%, a specificity of 64%, a PPV of 46.9%, and an NPV of 85.4%.

**Table 4:** Results of univariate and multivariate analyses of serological parameters by clinical outcomes in 105 patients with chronic hepatitis.

	Progression to cirrhosis				HCC				Liver decompensation				Liver-related death			
	(31 patients)				(10 patients)				(7 patients)				(7 patients)			
	Univariate		Multivariate		Univariate		Multivariate		Univariate		Multivariate		Univariate		Multivariate	
	OR	p	OR	p	OR	p	OR	p	OR	p	OR	p	OR	p	OR	p
**HDV-** **RNA**	1.57	<0.001	1.56	0.005	1.66	0.033	2.64	0.025	1.65	0.074	2.01	0.083	1.44	0.144	1.82	0.095
	(1.20–2.05)		(1.14–2.12)		(1.04–2.65)		(1.13–6.16)		(0.95–2.86)		(0.91–4.43)		(0.88–2.33)		(0.90–3.68)	
**HBV-** **DNA**	1.00	0.983	1.09	0.580	0.95	0.801	1.29	0.410	0.95	0.831	1.06	0.843	1.03	0.913	1.19	0.5555
	(0.77–1.29)		(0.80–1.50)		(0.62–1.44)		(0.70–2.37)		(0.58–1.56)		(0.60–1.86)		(0.65–1.62)		(0.67–2.09)	
**HBsAg** *	1.26	0.385	0.97	0.939	0.91	0.802	0.33	0.094	0.79	0.552	0.49	0.269	0.70	0.360	0.37	0.099
	(0.75–2.10)		(0.50–1.89)		(0.45–1.87)		(0.09–1.21)		(0.36–1.73)		(0.14–1.73)		(0.33–1.49)		(0.12–1.2)	

Multivariate models included HDV-RNA, HBV-DNA, HBsAg, age, sex, alcohol consumption, HBeAg and IFN.

*Four patients with missing values.

## Discussion

This study investigates a large cohort of patients followed up for a median period of 9.5 years, selected from a wider cohort already published [8]. The only criteria for selection was the availability of multiple frozen serum samples collected at baseline as well as during follow-up, in the respect of the overall characteristics of the original population. The study provides novel insights into the role and reciprocal interactions of HBV and HDV affecting the clinical outcome of HDV-related chronic liver disease. As cirrhosis is the major risk factor for late clinical complications, the non-cirrhotic patients with histological signs of chronic hepatitis alone, and those with histologically or clinically proven cirrhosis were analysed, whereas the levels of HDV RNA, HBV DNA and HBsAg were analysed in all of the samples collected at the time of clinical presentation, as well as in the samples collected from patients at the time of any subsequent development of cirrhosis or occurrence of any major event like HCC, clinical decompensation and liver related death.

The main finding of the study was that the clinical significance of HDV viremia is different in patients with chronic hepatitis alone and in those with established cirrhosis. Indeed, the univariate and multivariate analyses showed that the level of HDV viremia was the only virological parameter related to progression to cirrhosis and the end-stage complications of HDV, including HCC and clinical decompensation, in the patients who were not cirrhotic at the time of clinical presentation. This is the first study showing that the level of HDV viremia is the major driver of disease progression in patients with chronic hepatitis D, although our results are in line with previous findings that persistent HDV replication is the only predictor of an adverse outcome in patients with hepatitis delta [8]. Given the well-known inhibitory effect of HDV on HBV replication [21], [22], it is not surprising that most of the circulating infectious viral particles contain HDV RNA instead of HBV DNA. In a study of patients with HBV infection alone whose design was similar to that of ours, Iloeje *et al*. found a close correlation between progression to cirrhosis and the level of circulating virus measured at the time of the first clinical observation, after censoring for important comorbidity factors such as age, gender, smoking and alcohol abuse [23].

Interestingly, we found similar baseline HDV RNA levels in our cirrhotic and non-cirrhotic patients, which suggests that, in addition to only predicting progression in the long term, quantifying HDV viremia has no value in cross-sectional evaluations. This hypothesis is supported by a recent cross-sectional analysis of 80 patients from different countries that showed no correlation between HDV RNA levels and histological necro-inflammation and fibrosis scores [11].

Another interesting finding of our study is that, once cirrhosis has developed, HDV RNA levels lose any significant relationship with disease progression, i.e. the occurrence of HCC, decompensation and liver related death. This finding may appear controversial from our previous results. As a matter of fact, in the previous publication we indicated that persistent HDV RNA over time was a risk factor associated to the occurrence of clinical decompensation, regardless from quantitative levels of HDV RNA. We found differences in terms of median follow-up, having chronic hepatitis patients significant longer observation time. One possible explanation is that for each patient we considered the first available sample as the baseline observation, regardless from the first evidence of liver disease. A previous study [24] has found that HDV viral replication tends to decline at the most advanced stages of liver disease, whereas serum HBV DNA levels may increase and, together with our data, this suggests that some factors not included in the present analysis, including comorbidities, lifestyles and genetic predisposition, may influence the onset of complications in cirrhotic patients. This interpretation is supported by our finding that serum HDV RNA is independently associated with progression to cirrhosis and the development of HCC in patients with chronic hepatitis alone.

When we analysed the serological variables against the risk of developing at least one unfavourable event like progression to cirrhosis, HCC, clinical decompensation or liver-related death in these patients, there was a clear-cut trend towards a quantitative relationship between viremia and the risk of liver complication. A HDV RNA levels of >600,000 copies/mL predict in our cohort a risk of cirrhosis in about 50% of patients with chronic hepatitis (PPV = 46.9%) whereas lower values are a useful means of excluding the development of cirrhosis in a large number of cases (NPV = 85.4%). One possible explanation for higher levels of HDV RNA being associated with a greater risk of disease progression in patients with chronic hepatitis delta, is the influence of virus replication on cell immune responses against HDV-infected liver cells, as recently suggested by studies of host T cell immune responses in hepatitis delta genotypes 1 and 2 [25], [26]. The possibility that genetic diversity of HDV plays a role in hepatitis progression is less likely since in our cohort of mainly Italian patients (95%), HDV genotype 1 was the most prevalent infecting strain [27].

While our findings clearly need to be validated in prospective studies of larger patient populations, still they underline the importance of treatment before cirrhosis occurs because, once it has developed, our results indicate that there is no association between any of the serological parameters and the occurrence of at least one unfavourable event, like the development of HCC, liver decompensation or liver-related death.

We did not find any direct or indirect correlation between HDV RNA and HBV DNA levels. As HDV proteins p24 and p27 repress the HBV enhancer by inhibiting HBV replication, and HDV p27 transactivates the interferon alpha-inducible MxA gene, which also inhibits HBV replication [28], it was no surprise to find that most of our patients had no detectable HBV. The same was found in long-term clinical study of potentially highly viremic HBeAg positive patients with chronic hepatitis delta [29], which demonstrated that most of the patients had HBV DNA levels of <200,000 IU/mL at least once during follow-up, and 60% had levels of <2,000 IU/mL. It has recently been found that HDV patients carrying mutations at nucleotide positions 1762, 1764 and 1896 in the basal core promoter/precore region also have low HBV levels [30].

Consistent with the epidemiology of HDV in the Mediterranean basin, only 8% of the patients in our cohort were HBeAg positive at baseline, and there was no significant correlation with clinical outcome, as previously reported [29]. However, our findings and those from Spain [31] conflict with a recent finding from Turkey of the existence of a correlation between serum HBsAg levels and HDV viremia, regardless of histological staging and the grading of liver disease in patients with chronic hepatitis delta [11].

In conclusion, chronic hepatitis D is highly likely to progress to cirrhosis and HCC in Italy, and this propensity is greater the higher the level of HDV viremia at the time of presentation. However, once cirrhosis has developed, the role of high HDV levels as a predictor of a negative outcome becomes attenuated.
